# Reptile-associated Salmonellosis in Man, Italy

**DOI:** 10.3201/eid1202.050692

**Published:** 2006-02

**Authors:** Marialaura Corrente, Marta Totaro, Vito Martella, Marco Campolo, Alessio Lorusso, Massimo Ricci, Canio Buonavoglia

**Affiliations:** * University of Bari, Bari, Italy;; † Agenzia Regionale Protezione Ambiente Puglia, Brindisi, Italy

**Keywords:** Salmonella, reptiles, man, Italy, letter

**To the Editor:** Reptiles are reservoirs of a wide variety of *Salmonella* serotypes, including all S*almonella enterica* subspecies and *S*. *bongori*. In reptiles born in captivity or kept as pets, *S. enterica* subsp. *enterica* is frequently isolated (*1*). *Salmonella* strains are well adapted to reptiles, and they usually cause asymptomatic infections in such animals, while retaining pathogenicity for warm-blooded animals. For several years, reptiles have been recognized as a source of human salmonellosis. In North America, reptile-associated salmonellosis (RAS) has been reported, particularly in children, the elderly, or immunocompromised persons; severe and fatal infections are described occasionally (*2*). In contrast, only a limited amount of information on RAS is available in Europe. We report a case of RAS that occurred in an adult man in Italy.

A 32-year-old man had symptoms of enteritis. For 2 weeks, he had experienced intermittent watery diarrhea, mild fever, and abdominal pain. He was then treated with ciprofloxacin, and after 15 days of treatment, he recovered from enteritis. A stool sample, collected before treatment, underwent bacteriologic analysis, and *Salmonella* spp. were identified biochemically (api 20E, bioMérieux, Marcy l'Etoile, France) and by a polymerase chain reaction assay specific for the *invA* gene of *Salmonella* spp (*3*). Since the man was a reptile owner, RAS, rather than a foodborne infection, was initially suspected. He owned several cold-blooded animals; all had been tested for *Salmonella* spp. (at least 3 times at 2- to 3-week intervals), and results were negative. Three weeks before the onset of enteric symptoms, he acquired a boa (*Boa imperator*) that was subjected to routine analysis for *Salmonella* spp. in our laboratories (*1*). *Salmonella* spp. were isolated from a cloacal swab of the snake. Subsequently, both the human and reptile *Salmonella* isolates were characterized as *S*. *enterica* serovar Paratyphi B. In addition, both strains were found to be d-tartrate–fermenting (dT+) biovars (*4*), susceptible to ampicillin, amoxicillin-clavulanic acid, cephalothin, ceftazidime, gentamicin, streptomycin, chloramphenicol, tetracycline, neomycin, nalidixic acid, norfloxacin, and ciprofloxacin and resistant to sulfamethoxazole and co-trimoxazole.

By pulsed-field gel electrophoresis analysis of DNA, the strains displayed the same pattern, which suggests a clonal origin (*4*). The isolates were also assayed for virulence-associated genes. The *Sop*E1 gene was detected in both isolates, and the *avr*A gene was not detected, which is consistent with an invasive pathovar of *S*. Paratyphi B (*4*). Conversely, the *spv*C, *pef*, and *sef* genes were not detected (*5*).

In recent years, a general increase in RAS detection has been observed, which may be the result of the increasing diffusion of reptiles as pets and a better awareness of RAS risk. In the United States, annual reports of RAS cases are published by the Centers for Diseases Control and Prevention (*2*). In Europe, studies on free-living and captive reptiles have shown a high prevalence of *Salmonella* spp (*1*). Nevertheless, national surveillance systems for RAS do not exist, and epidemiologic data are incomplete.

Notably, since Sweden became a member of the European Union in 1995, and the import restriction rules for reptiles were removed, a marked increase in RAS was observed in that country (*6*). As the deregulation of the trade in reptiles is applied, in agreement with the European Union laws, a similar scenario may be projected in other European countries. As is the case for nontyphoid salmonellosis, RAS may be underestimated, especially if patients are not hospitalized. Although a few cases of RAS have been previously reported in children in Italy (*7**,**8*), this report provides the first description of RAS in adults. *S*. Paratyphi B dT+, also known as *S. enterica* serovar Java, has been isolated in reptiles and tropical fish and has been associated with epidemics of human salmonellosis acquired from food, such as goat milk or chicken (*9*). The evidence shows that salmonellosis by *S*. Paratyphi B dT+ apparently occurs more frequently in adults (*10*), while so-called exotic reptile strains seem to be more prone to causing salmonellosis in children (*7**,**8*), which has led to the proposition that *S*. Paratyphi B dT+ strains may be highly pathogenic. By screening virulence-associated genes, both our isolates were found to be *Sop*E1+ and *avr*A–, a pattern usually observed in the systemic pathovars of *S*. Paratyphi B (*4*) and associated with invasiveness, which suggests a high pathogenic potential. Accordingly, strict preventive sanitation measures should be adopted when handling reptiles (*2*), and reptiles should be always regarded as a potential source of pathogenic *Salmonella* strains for humans.
